# Comprehensive analysis of the prognostic value and immune infiltration of *FGFR* family members in gastric cancer

**DOI:** 10.3389/fonc.2022.936952

**Published:** 2022-09-06

**Authors:** Chengcheng Yang, Dingli Song, Fengyu Zhao, Jie Wu, Boxiang Zhang, Hong Ren, Qi Sun, Sida Qin

**Affiliations:** ^1^ Department of Oncology, The First Affiliated Hospital of Xi’an Jiaotong University, Xi’an, China; ^2^ Department of Thoracic Surgery, The First Affiliated Hospital of Xi’an Jiaotong University, Xi’an, China; ^3^ Department of General Surgery, The First Affiliated Hospital of Xi’an Jiaotong University, Xi’an, China

**Keywords:** fibroblast growth factor receptors (FGFRs), gastric cancer, immune cell infiltration, prognosis, anti-programmed cell death 1 monoclonal antibodies (Anti-PD-1 mAB), database analysis

## Abstract

**Background:**

Fibroblast growth factor receptors (*FGFRs*) modulate numerous cellular processes in tumor cells and tumor microenvironment. However, the effect of *FGFRs* on tumor prognosis and tumor-infiltrating lymphocytes in gastric cancer (GC) remains controversial.

**Methods:**

The expression of four different types of *FGFRs* was analyzed *via* GEPIA, TCGA-STAD, and GTEX databases and our 27 pairs of GC tumor samples and the adjacent normal tissue. Furthermore, the Kaplan–Meier plot and the TCGA database were utilized to assess the association of FGFRs with clinical prognosis. The R software was used to evaluate *FGFRs* co-expression genes with GO/KEGG Pathway Enrichment Analysis. *In vitro* and *in vivo* functional analyses and immunoblotting were performed to verify *FGFR4* overexpression consequence. Moreover, the correlation between *FGFRs* and cancer immune infiltrates was analyzed by TIMER and TCGA databases. And the efficacy of anti-PD-1 mAb treatment was examined in NOG mouse models with overexpressed *FGFR1* or *FGFR4*.

**Results:**

The expression of *FGFRs* was considerably elevated in STAD than in the normal gastric tissues and was significantly correlated with poor OS and PFS. ROC curve showed the accuracy of the *FGFRs* in tumor diagnosis, among which *FGFR4* had the highest ROC value. Besides, univariate and multivariate analysis revealed that *FGFR4* was an independent prognostic factor for GC patients. According to a GO/KEGG analysis, the FGFRs were implicated in the ERK/MAPK, PI3K-AKT and extracellular matrix (ECM) receptor signaling pathways. *In vivo* and *in vitro* studies revealed that overexpression of FGFR4 stimulated GC cell proliferation, invasion, and migration. In addition, *FGFR1* expression was positively correlated with infiltrating levels of CD8+ T-cells, CD4+ T-cells, macrophages, and dendritic cells in STAD. In contrast, *FGFR4* expression was negatively correlated with tumor-infiltrating lymphocytes. Interestingly, overexpression of *FGFR1* in the NOG mouse model improved the immunotherapeutic impact of GC, while overexpression of *FGFR4* impaired the effect. When combined with an *FGFR4* inhibitor, the anti-tumor effect of anti-PD-1 treatment increased significantly in a GC xenograft mouse model with overexpressed *FGFR4.*

**Conclusions:**

*FGFRs* has critical function in GC and associated with immune cell infiltration, which might be a potential prognosis biomarker and predictor of response to immunotherapy in GC.

## Introduction

Gastric cancer (GC) has been described as one of the most common cancers, and the fourth leading cause of cancer-related mortality (1). Unfortunately, diagnosis is often in the advanced stage when only palliative treatment is available. Despite advances in treatment, including surgery, radiation, chemotherapy, and immunotherapy, the prognosis remains poor. The 5-year overall survival (OS) rate of the Stage IV GC is less than 10% (2). Therefore, more innovative agents should be developed to improve patient prognosis.

The mammalian fibroblast growth factor receptor (FGFR) family includes four highly conserved receptors (*FGFR1*, *FGFR2*, *FGFR3*, and *FGFR4*) *(* 3). They are single-pass transmembrane proteins typically containing an extracellular domain, a transmembrane domain, and an intracellular tyrosine kinase domain (4). Fibroblast growth factors (FGFs) bind to their receptors and subsequently dimerized with the receptor. Then the dimerizer triggers a cascade of intracellular processes that activate crucial survival and proliferation signaling pathways. Irregular FGFR signaling pathway are associated with various physiological process for many tumor types, including oncogenesis, tumor progression, and resistance to anti-cancer treatments (5–7). *FGFR1* is amplified in an estimated 10% of breast cancers and 12% of non-small cell lung cancer (NSCLC), leading to a poor patient prognosis (8–9). According to a recent study, mutations in the gene encoding FGFR3 are more prominent in luminal 1 bladder tumors (10). *FGFR4* has been implicated in hepatocarcinogenesis (11). Multiple studies showed genetic aberration of *FGFR2*, thus serving as a diagnostic biomarker and therapeutic target for GC (6, 12). Therefore, many inhibitors have been developed against specific FGFRs, with some as candidates for potential pharmaceutical therapy. However, most FGFR inhibitors have shown only a modest effect in GC during clinical trials although the data from preclinical study were highly promising (13, 14). Thus, extensive studies on the *FGFRs* are necessary to find pharmacological targets for GC therapy.

The potential use of immunotherapy in GC has received considerable interest in recent decades. CHECKMATE-649 clinical trials showed the great clinical value of Nivolumab (Opdivo) in the late-stage GC patients and elevated survival from 11.1 months to 14.4 months in Combined Positive Score (CPS) >5 population (15). Although Epstein-Barr virus, microsatellite instability-high (MSI-H), and tumor mutational burden was used as indicators to predict the prognosis of GC (16), there is still only a subset of patients that benefit from PD-1/PD-L1 checkpoint blockade. Thus, it is critical to identify more effective immunotherapy targets and predictive biomarkers. Previous experiments have found that *FGFR* is associated with immunity (17, 18), but the correlation between FGFR protein expression and tumor-infiltrating immune cells is not clear. In addition, the underlying mechanism how *FGFRs* regulates the immune environment and their association with the efficiency of immunotherapy remains unclear.

The dysregulated expression levels of *FGFRs* and their association with clinicopathological characteristics have been described in cancers, such as NSCLC, gallbladder, hepatic carcinoma, and breast cancer. However, the function and prognostic role of distinct FGFR family members in GC remains controversial. Moreover, the mechanisms of *FGFRs* in tumorigenesis and tumor immunology are still unclear. This study analyzed the expressions of four types of *FGFRs* in different databases and patients with GC and verified the results through *in vivo* and *in vitro* studies. We aimed to find the expression patterns, potential functions, distinct diagnosis, and prognostic values of *FGFRs* in GC. More importantly, we further investigated the correlation of FGFRs with tumor-infiltrating immune cells and evaluated the potential predictive value of *FGFRs* to immunotherapy response through database analysis and *in vivo* experiments.

## Materials and methods

### Data source

The RNA-sequencing profile for 375 primary GC, 32 normal gastric tissues, and their corresponding clinicopathological characteristics (age, grade, stage, and sex) and survival time were download from The Cancer Genome Atlas-Stomach Adenocarcinoma (TCGA-STAD) database (https://portal.gdc.cancer.gov/). Similarly, the profile of 100 normal gastric tissues and 300 GC tumor were download from GEO database (GSE66229).

### Reagents

Anti-PD-1 mAb (Nivolumab) was obtained from Bristol-Myers Squibb Company, New York, New York, USA. FGF 401 (Selleck Chemicals, Houston, Texas, USA), FGFR1(D8E4, #9740), FGFR4(D3B12, #8562), Erk1/2(137F5, #4695), Phospho- Erk1/2 (Thr202/Tyr204) (D13.14.4E, #4370), Akt (C67E7, #4691), Phospho-Akt (Ser473) (D9E, #4060), E-Cadherin (4A2, #14472), MMP-9 (D6O3H, #13667) antibody (Cell Signaling Technology, Danvers, Massachusetts, USA), anti-fibronectin (ab32419), anti-MMP2 (ab97779) (Abcam, Cambridge, UK), and β-actin (Sigma-Aldrich, St Louis, Missouri, USA) were used as obtained.

### Patients

Twenty-seven pairs of GC tissues and their adjacent normal tissues were obtained from General Surgery Department of the First Affiliated Hospital at Xi’an Jiaotong University, China, in 2020 and 2021. Immediately after resection, GC and normal adjacent tissues specimens were stored at -80°C. This study was approved by the Ethics Committee of the First Affiliated Hospital of Xi’an Jiaotong University, and conducted in accordance with the Declaration of Helsinki principles. Informed consent was obtained from all patients.

### Plasmids and stable cell lines establishment

All human GC cell lines were obtained from the Cell Resource Center, Peking Union Medical College (Peking, China) and cultured in Roswell Park Memorial Institute Medium (RPMI) or Dulbecco’s Modified Eagle Medium (DMEM) with 10% Fetal Bovine Serum (Invitrogen) at 37°C and 5% CO_2_. The 293T cells were obtained (Clontech Laboratories, San Francisco, California, USA) and cultured in DMEM with high glucose. pHAGE-FGFR1 and pHAGE-FGFR4 were gifts from Gordon Mills & Kenneth Scott, Addgene plasmid #116740, #116743. Retroviral infections were performed as previously described (19, 20). Transfected cells were established under puromycin selection, until stable cell lines were generated as N87-EV, N87-FGFR1, and N87-FGFR4.

### The differential expression analysis of *FGFR* family in GC

Gene Expression Profiling Interactive Analysis database (GEPIA, gepia.cancer-pku.cn) provided vast integrated genome expression profiles in GC and normal samples (21). The mRNA expression levels of *FGFR* in GC tissues were compared to normal tissues. We further examined the expression of *FGFRs* in multiple tumor samples through ONCOMINE (www.oncomine.org) and Tumor Immune Estimation Resource (TIMER) databases (https://cistrome.shinyapps.io/timer/) to validate the former result. In addition, we used the Cancer Genome Atlas (TCGA) and Genotype-Tissue Expression (GTEX) data to evaluate the expression of *FGFRs* members in tumor and adjacent normal tissues to explore the expression in GC patients of different clinicopathological characteristics. The data was analyzed through the “ggplot2” R package using R software from Bell Laboratories (formerly AT&T, now Lucent Technologies, New Jersey, USA) by John Chambers and colleagues.

### Survival analysis

Kaplan–Meier plot (www.kmplot.com) was utilized to examine the prognosis of *FGFRs* expression in STAD. The survival outcomes were OS and progress-free survival (PFS) (22). In addition, the association were evaluated between mRNA levels of *FGFRs* and clinical characteristics. The univariate and multivariate analysis were applied using the Cox regression model. A *p*-value<0.05 was considered significant.

### Enrichment analysis of *FGFRs* co-expression network in GC

The LinkedOmics database was used to analyze the correlated genes with *FGFRs* through the “limma” R package to extract the expression profiles of the correlated gene in TCGA-STAD data sets. Then, we drew the correlation heatmap of the top 10 genes through TCGA based on *FGFRs* different expression groups (Pearson Correlation Coefficient, *p*<0.05). The Gene Ontology (GO) and Kyoto Encyclopedia of Genes and Genomes (KEGG) enrichment analyses were performed using the R package “clusterProfiler” to assess the relative biological functions and molecular pathways regulated by the correlated genes. A *p*-value<0.05 was set as significant for screen criteria.

### *FGFRs* genomic alterations

The cBioPortal database (http://cbioportal.org) was used to explore the *FGFRs* gene alterations in GC (23). Then the TIMER somatic copy number alternation (SCNA) module compares tumor infiltration levels in tumors with different SCNA for FGFR family members through a two-sided Wilcoxon rank-sum test.

### Immune cell infiltration analysis

TIMER database is a publicly available source for evaluating the impact of different immune cells in diverse cancers (24). The correlation between *FGFRs* and the 24 different kinds of immune cell infiltrates in gastric cancer samples, including CD8+ T-cells, CD4+ T-cells, neutrophils, macrophages, and natural killer cells, was achieved by the “ssGSEA” method through the GSVA package with R computing. TIMER was utilized to assess the correlation between *FGFRs* expression and tumor purified immune cell infiltration.

### Functional experiments, real-time quantitative PCR, and immunoblotting

Functional experiments (cell proliferation, transwell invasion assay, and scratch assay), RT-qPCR, and immunoblotting were performed as described in our previous articles (25–26). *FGFR1, FGFR2, FGFR3, and FGFR4* primers (OriGene, Rockville, Maryland, USA) were used to detect mRNA levels by real-time quantitative reverse transcription polymerase chain reaction (RT- PCR). All the experiments were repeated three times independently.

### *In vivo* studies in humanized NOG mouse tumor model

Immunodeficient NOD Cg-Prkdc^scid^Il2rg^tm1Sug^/JicCrl (NOG) mice (Weitong Lihua Experimental Animal Co., Ltd, Beijing, China) were used to receive human immune cells and established a humanized immune system. Peripheral blood mononuclear cells (PBMCs) from healthy donors were isolated and 2×10^7^ cells were administered intraperitoneally into the mice. BALB/c nude mice were purchased from the Laboratory Animal Center of Xi’an Jiaotong University. N87-EV, N87-FGFR1, and N87-FGFR4 cells (5 ×10^6^) were administered subcutaneously into mice. After 10 days, the subcutaneous tumors had grown to a size that could be measured (approximately 90 mm^3^). Then FGF401was diluted in 6% 1M HCl/100 mM citrate buffer pH 2.5 (citrate salt) and administered intragastrically at 30 mg/kg daily. Mouse was injected with Anti-PD-1 mAb (nivolumab) at 200 mg/mouse twice a week for 4 weeks. Every 2 days, the tumor volume and body weight were measured and calculated as (length × width^2^)/2. Mice were euthanized when Tumor volume (TV) exceeded 1,500 mm^3^ or when the mouse’s weight decreased by < 70% of that at Day 1. Survival (in days) was defined as the time for each mouse from Day 0 until the day of death or euthanization. Survival curve was built for each group through GraphPad Prism 7.0 (GraphPad Software, San Diego, California, USA). The mice experiments were performed following the protocols approved by the by the Ethics Committee of the First Affiliated Hospital of Xi’an Jiaotong University.

### Statistical analysis

All results were statistically analyzed and summarized as a mean and standard error of measurement (SEM) by GraphPad Prism Software. The two-tailed Student’s t-test was applied to assess the differences between the groups. Spearman’s correlation test was performed to determine the correlation between groups. *p* ≤ 0.05 was considered statistically different.

## Results

### Distinct expression of *FGFRs* in GC patients

**Figure 1:**
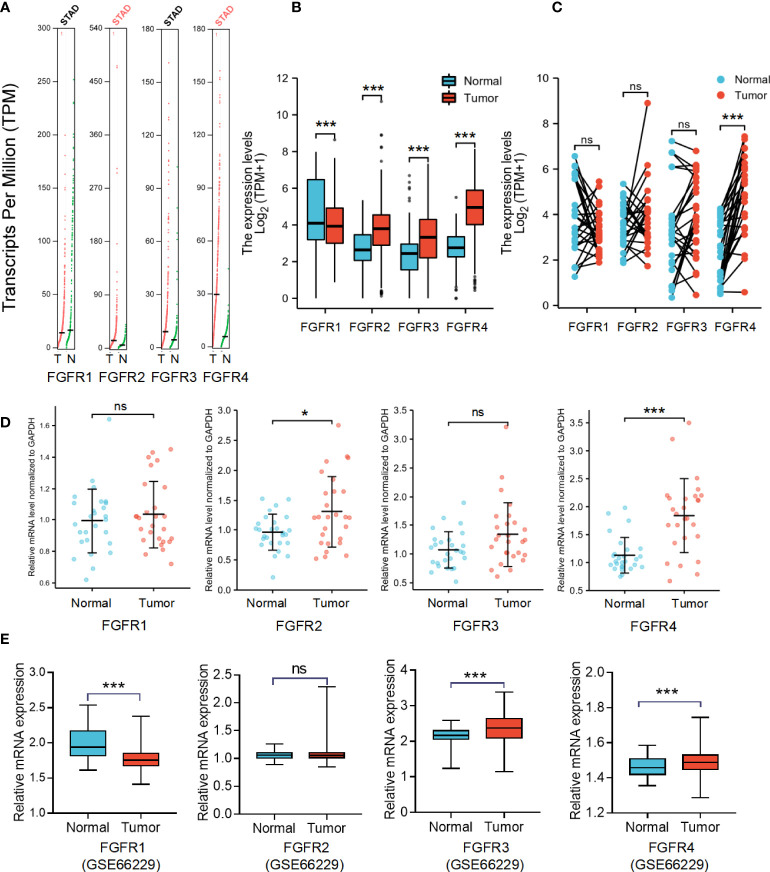
The mRNA expression levels of *FGFRs* in GC. **(A)** The differential expression of *FGFR* family members in cohort tumor and non-tumor tissues was analyzed with GEPIA. **(B)** The difference in expression of *FGFRs* between STAD in TCGA data sets and normal gastric tissues in GTEX data sets. **(C)** The differences in expression of *FGFRs* between STAD and matched normal tissues from TCGA-STAD database were determined by GEPIA. **(D)** The mRNA expression of *FGFRs* in gastric tumor tissues and matched adjacent non-tumor tissues from our samples. **(E)** The differential expression of *FGFRs* between tumor and matched normal tissues from GSE66229 database. *p < 0.05; ***p < 0.001; **** *p* <0.0001; ns, not significant; GC, gastric cancer; T, tumor; N, normal.

To determine differences in *FGFR* family expression between tumor and normal tissues, we analyzed the mRNA level both in various datasets and our GC samples. First, the differential expression of *FGFR* family members was evaluated with pan-cancer analysis with Oncomine and TIMER databases, which revealed that the expression of *FGFR* family in lung cancer, breast cancer, liver cancer, colorectal cancer, head-neck cancer, GC, and sarcoma was higher than that in normal tissues (**Figure S1**). Then the transcriptional levels of *FGFR* family in GC and their adjacent normal tissues were evaluated in the GEPIA database (**Figure 1A**). According to the result, *FGFR2* and *FGFR4* expression were considerably increased in tumors than that in normal tissue. But there was no significant difference between the tumors and normal tissues in *FGFR3* and *FGFR1*. Next, we downloaded the original files from TCGA-STAD and GTEx database to analyze *FGFR1-4* expression. In this database, the expression levels of *FGFR2*, *FGFR3*, and *FGFR4* were remarkably higher in STAD than that in normal tissues, while *FGFR1* was significantly lower (**Figure 1B**). Furthermore, the analysis of paired samples from the TCGA database suggested that only *FGFR4* expression was significantly elevated in tumor samples (**Figure 1C**). To further ensure the accuracy of results we analyzed above, we examined the mRNA level in 27 pairs of GC tissues and their adjacent normal tissues by qRT-PCR. The results indicated that the RNA level of *FGFR2* (*p*<0.05) and *FGFR4* (*p*<0.001) was considerably higher in tumor tissues compared to the matched adjacent non-tumor tissues (**Figure 1D**). No significant difference was found between tumor and adjacent normal tissues in the mRNA level of *FGFR1* and *FGFR3*. Moreover, we further validated the expression of FGFRs in another GSE66229 database. The expression of *FGFR3* and *FGFR4* were significantly higher in tumor than in normal samples in GC (*p*<0.001) (**Figure 1E**).

### The prognostic and diagnostic value of *FGFRs* in GC

**Figure 2:**
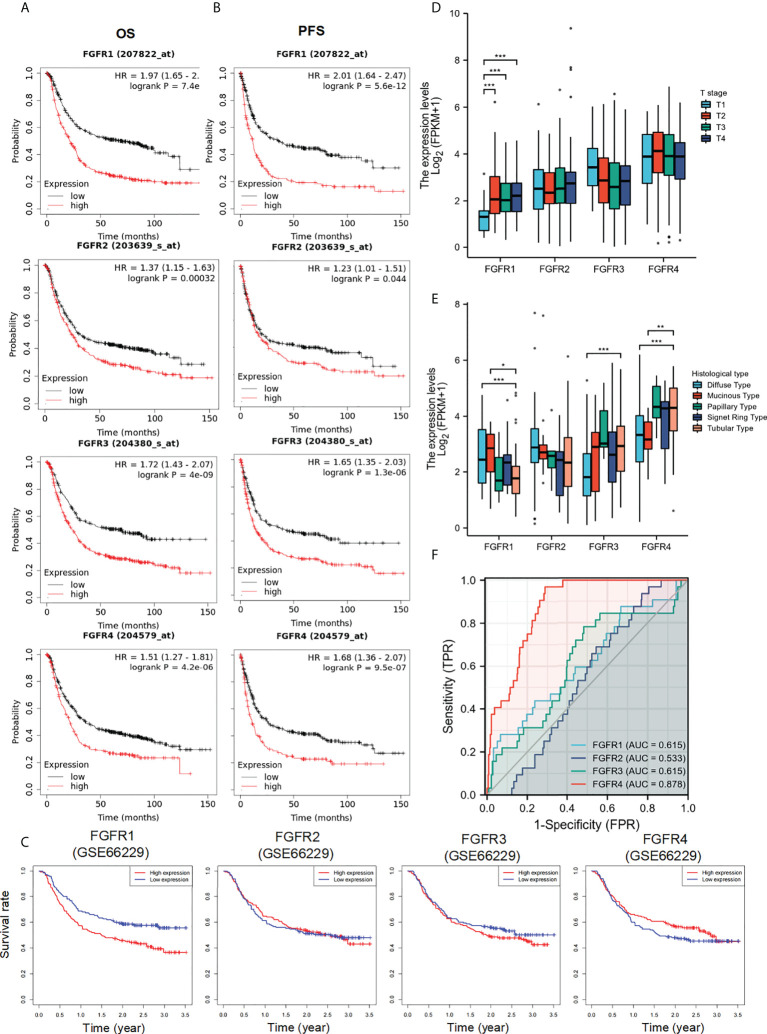
The diagnostic and prognostic value of *FGFRs* in GC. **(A, B)** The OS **(A)** and PFS **(B)** survival curves of *FGFRs* in TCGA-STAD in Kaplan–Meier plot databases. **(C)** The OS survival curves of FGFRs in GSE66229 database. **(D, E)** The expression of *FGFRs* in different T stages **(D)** and histologies **(E)** were analyzed in TCGA-STAD data sets. **(F)** ROC curve analysis of *FGFRs* in the diagnosis of GC. *p < 0.05; **p < 0.01; ***p < 0.001; OS, overall survival; PFS, progression-free survival; GC, gastric cancer.

To further assess the prognostic and diagnostic value of *FGFRs* in STAD, we analyzed the impact of *FGFR* family expression on survival using the Kaplan–Meier plot. High expression mRNA levels of the whole FGFR family were significantly associated with OS and PFS in GC patients (**Figures 2A, B**). The result from GSE66229 database showed the same trend. The expression of *FGFR1* and *FGFR4* were also correlated with the poorer overall survival in GC. But FGFR2 and FGFR3 (**Figure 2C**). Furthermore, we analyzed the correlation between FGFRs and GC patients’ characteristics and found *FGFR1* expression was substantially associated with the T stage, whereas other clinical features were not markedly different with high expression of *FGFR* (**Figure 2D**, **Figure S2**). *FGFR1*, *FGFR 3*, and *FGFR 4* expression varied in different histological types, among which the difference between diffuse and tubular tumors was most pronounced (**Figure 2E**). Then, we assessed the correlation between *FGFR* family expression and the clinicopathological features with univariate and multivariate Cox regression analysis using TCGA data. As shown in **Table 1**, N stage, distant metastasis, age, and *FGFR4* were independent prognostic factors for GC patients, while *FGFR1*, *FGFR2*, and *FGFR3* had no significant association with an increased risk of GC and poor OS and PFS in univariate or multivariate analyses. Additionally, the ROC analysis revealed that the *FGFRs* expression levels had high diagnostic potential for GC, among which *FGFR4* was the most accurate (*FGFR1*: AUC: 0.615, *FGFR2*: AUC: 0.533, *FGFR3*: AUC: 0.615, *FGFR4*: AUC: 0.878; **Figure 2F**).

**Table 1:** Univariate and multivariate COX risk model.

Characteristics	Total(N)	Univariate analysis		Multivariate analysis	
		Hazard ratio (95% CI)	*p*-value	Hazard ratio (95% CI)	*p-*value
T stage	362				
T1	18	Reference			
T2	78	6.725 (0.913–49.524)	0.061	5.528 (0.743–41.136)	0.095
T3	167	9.548 (1.326–68.748)	**0.025**	6.487 (0.887–47.464)	0.066
T4	99	9.634 (1.323–70.151)	**0.025**	5.786 (0.775–43.176)	0.087
N stage	352				
N0	107	Reference			
N1	97	1.629 (1.001–2.649)	**0.049**	1.277 (0.753–2.166)	0.364
N2	74	1.655 (0.979–2.797)	0.060	1.438 (0.833–2.480)	0.192
N3	74	2.709 (1.669–4.396)	**<0.001**	2.262 (1.338–3.826)	**0.002**
M stage	352	2.254 (1.295–3.924)	**0.004**	2.425 (1.326–4.435)	**0.004**
Age (≤65 *vs >*65)	367	1.620 (1.154–2.276)	**0.005**	1.811 (1.260–2.602)	**0.001**
*FGFR4*	370	1.397 (1.006–1.941)	**0.046**	1.431 (1.011–2.026)	**0.043**

### Enrichment analysis of *FGFRs* co-expression genes in GC

**Figure 3:**
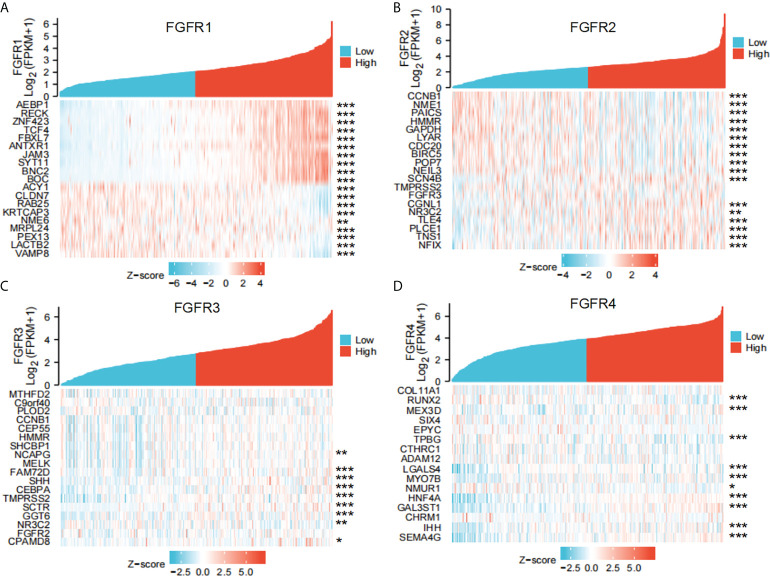
The co-expressed genes associated with the expression of *FGFRs* were analyzed as a heatmap by using the STAD data sets of TCGA. **(A)** FGFR1, **(B)** FGFR2, **(C)** FGFR3, **(D)** FGFR4. *p<0.05; **p<0.01; ***p<0.001.

**Figure 4:**
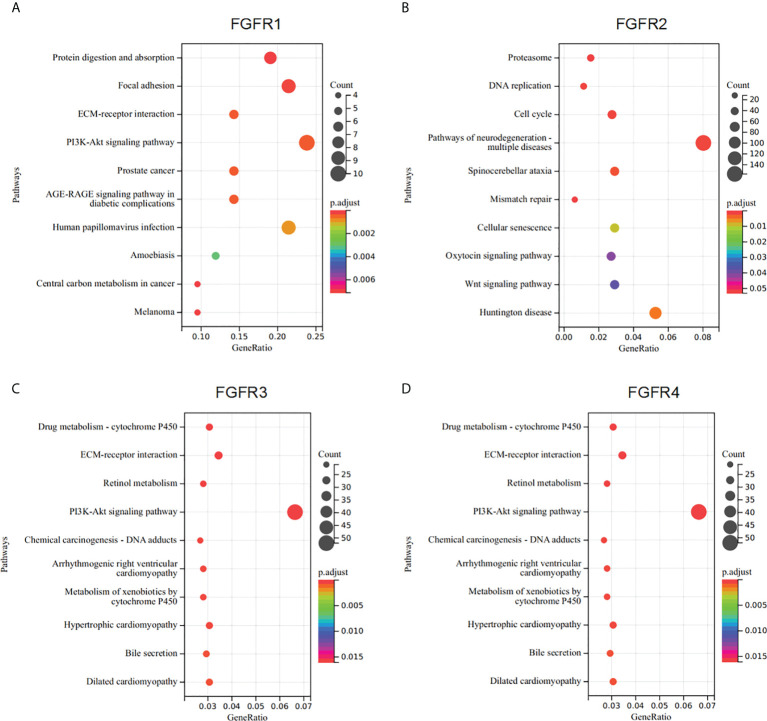
Enrichment analysis of *FGFRs* co-expression genes in GC. **(A–D)** The functions of genes significantly associated with *FGFR1* **(A)**, *FGFR2* **(B)**, *FGFR3* **(C)**, and *FGFR4* **(D)** alterations were predicted by the enrichment analysis of KEGG. GC, gastric cancer; KEGG, Kyoto Encyclopedia of Genes and Genomes.

To better understand the underlying mechanisms of *FGFRs* expression in GC, we evaluated the genes associated with the expression of FGFR family members in the STAD datasets of TCGA with the Limma R package. After the selection, upregulated and downregulated genes were identified according to the *FGFR* high and *FGFR* low expression group in STAD. a correlated heatmap was drawn between the different groups, which showed the highest ten positive and ten negative genes of every FGFR member (**Figures 3A–D**) (Pearson Correlation Coefficient, *p*<0.05). To verify the gene-gene interaction network, STRING and GeneMANIA were also used to explore the correlated genes. The potential target gene interactions with the FGFR family are shown in **Figure S3**. The interactions were generally in agreement with the genes from the association analysis. Then we performed a functional enrichment analysis to excavate the underlying biological function of FGFR based on the co-expressed genes. The KEGG analysis showed the *FGFR1/3/4* were closely related to the PI3K-AKT signaling pathway and ECM receptor interaction, while *FGFR2* was highly correlated with cell cycle, pathways of neurodegeneration multiple diseases, and Wnt signaling pathway (**Figures 4A–D**).

### *FGFR4* overexpression enhanced proliferation, invasion, and migration of GC *in vitro* and *in vivo*

**Figure 5:**
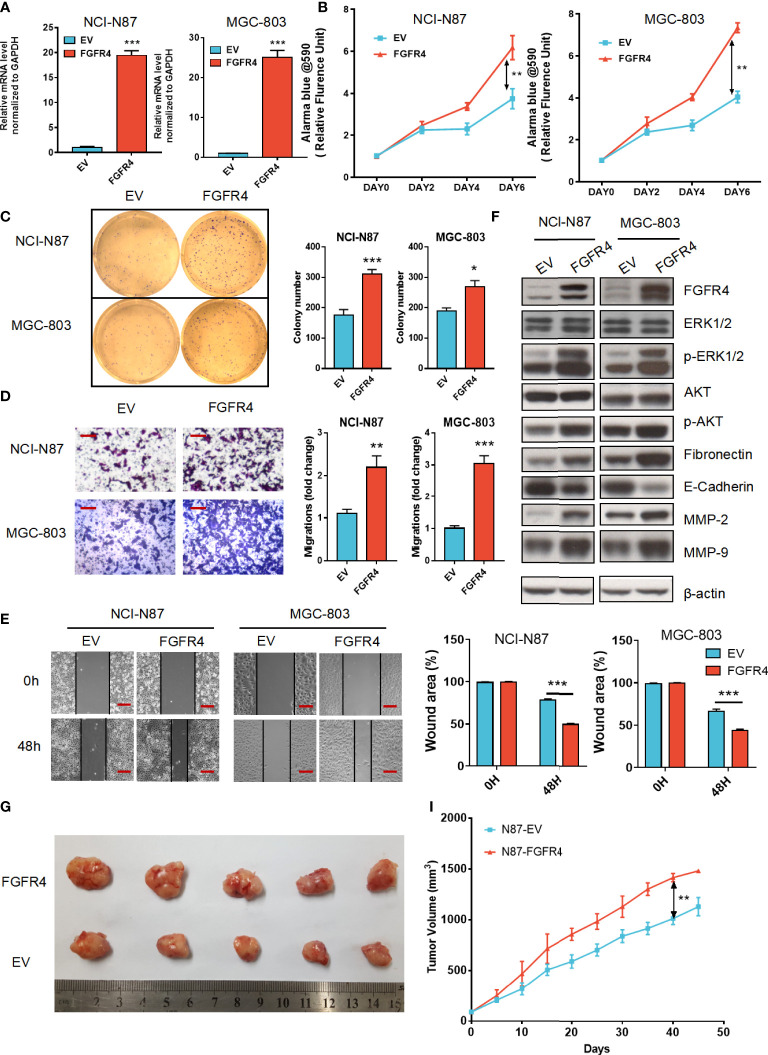
*FGFR4* overexpression in GC cells exhibits a tumor progression effect. **(A)** *FGFR4* mRNA levels were detected by qRT-PCR in NCI-N87 **(A)** and MGC-803 cells transfected with EV (control plasmid) and FGFR4 (p-HAGE -FGFR4). **(B)** *FGFR4* overexpression promoted cell proliferation in GC NCI-N87 and MGC-803 cells, respectively. **(C)** *FGFR4* overexpression improved the colony formation ability of GC cells. **(D, E)** Cell invasion and migration capacity were measured by the Transwell invasion assay **(D)** (scale bar, 100 μm) and Scratch assay **(E)** (scale bar, 200 μm). **(F)** N87-EV, N87-FGFR4, MGC803-EV, and MGC803-FGFR4 cells were collected with lysates subject to immunoblotting. **(G)** Representative images of nude mouse subcutaneous tumors for N87-EV and N87-FGFR4 cell lines. **(I)** Tumor volume was measured at the indicated time points. Data represent the mean of three independent experiments. *p < 0.05, **p < 0.01, ***p<0.001 student’s t-test; GC, gastric cancer; RT-PCR, real-time quantitative reverse transcription polymerase chain reaction; EV, empty vector.

In the results above, we found that *FGFR1*, *FGFR2*, *FGFR3*, and *FGFR4* were all associated with the prognosis of GC patients, in which *FGFR4* had the most prognostic and diagnostic value among the whole family. To further elucidate the functional role of *FGFR4* overexpression, NCI-N87 and MGC-803 cell lines with low *FGFR4* expression were transfected with lentivirus plasmid pHAGE-FGFR4. *FGFR4* overexpression stable cell line was established by puromycin selection. *FGFR4* overexpression was confirmed by qRT-PCR and immunoblotting (**Figures 5A, F**). The overexpression of *FGFR4* induced a significant elevation of cell proliferation according to the proliferation assay (**Figure 5B**). Similarly, plate colony formation showed considerably more colonies in the FGFR4 overexpressed group (**Figure 5C**). As to the transwell cell migration assay, high levels of FGFR4 resulted in the increase of both NCI-N87 and MGC-803 cells (**Figure 5D**). The data from scratch assay was consistent with the data above (**Figure 5E**). To ascertain the downstream regulation pathway of *FGFR4* overexpression, we examined the expression levels of representative markers in the ERK/MAPK pathway, PI3K/Akt pathway, and ECM signaling. Western blot analyses showed that p-ERK, p-AKT, fibronectin, and matrix metalloproteinase 2 (MMP-2) were remarkably elevated after *FGFR4* overexpression. Meanwhile, E-cadherin was significantly reduced in the N87-FGFR4 cells (**Figure 5F**, **Figure S4**). The data was consistent with the result from the KEGG pathway analysis and functional experiment. Moreover, the nude mice bearing *FGFR4* overexpression tumors (N87-FGFR4) showed better tumor-growth promotion than those with the control plasmid (**Figures 5G–I**). These results indicated that *FGFR4* overexpression promotes the malignancy and the EMT in GC. Therefore, *FGFR4* can also be expected to serve as a novel target for GC treatment.

### Genomic alterations of the *FGFR* family in GC

**Figure 6:**
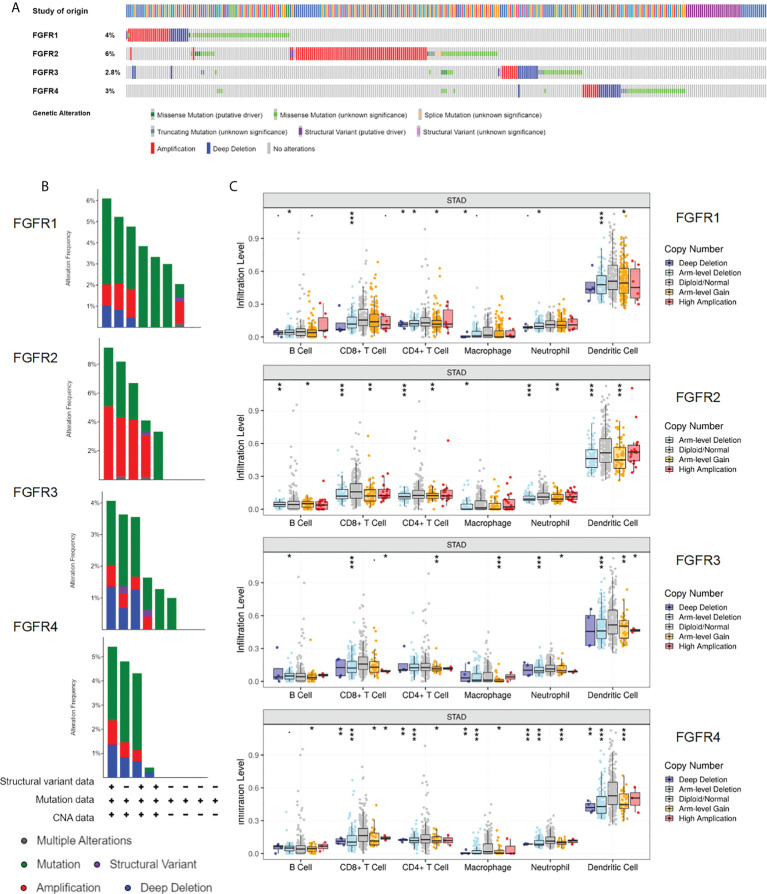
Genomic alterations of the FGFR family in GC. **(A, B)** The types and frequencies of *FGFR* family alterations in the GC samples through the cBioportal database. (Data base selected: TGCA, Nature 2014; TCGA, Firehose Legacy; TCGA, PanCancer Atlas; MSK,2020; UHK, Nat Genet 2011; Pifizer and UHK, Nat Genet 2014; U Tokyo, Nat Genet 2014; TMUCIH, PNAS, 2015. **(C)** Association between *FGFRs* gene copy number and immune cell infiltration levels in TCGA-STAD cohorts. *p < 0.05; **p < 0.01; ***p < 0.001; GC, gastric cancer.

Gene mutation was highly correlated with tumor origination and progression; therefore, we speculated that *FGFR* family variation in gene mutation might affect GC development. Using the cBioportal database, we initially investigated the types and frequencies of alterations in the *FGFR* family in GC samples. From the histogram, three types of gene alteration of *FGFR* family members were discovered in various GC cohorts, including mutation, deep deletion, and amplification. The mutation frequency of *FGFR2* was the most significant (6%), of which the majority were amplification mutations, whereas the three types of gene alterations were more balanced in *FGFR1*, *FGFR3*, and *FGFR4* (**Figures 6A, B**). However, in the survival analysis, no considerable differences were discovered between the *FGFR2* altered mutation group and the unaltered one (**Figure S5**). TIMER database was employed to further assess the association between the level of tumor infiltration and SCNA. The variations of *FGFRs* somatic copy number alterations were all significantly associated with immune cell infiltration levels of CD8+ T-cells, CD4+ T-cells, macrophages, and dendritic cells in GC, suggesting the involvement of *FGFRs* in regulating immune cells in the TME (**Figure 6C**).

### Expression of *FGFRs* related to immune cell infiltration in tumors

**Figure 7:**
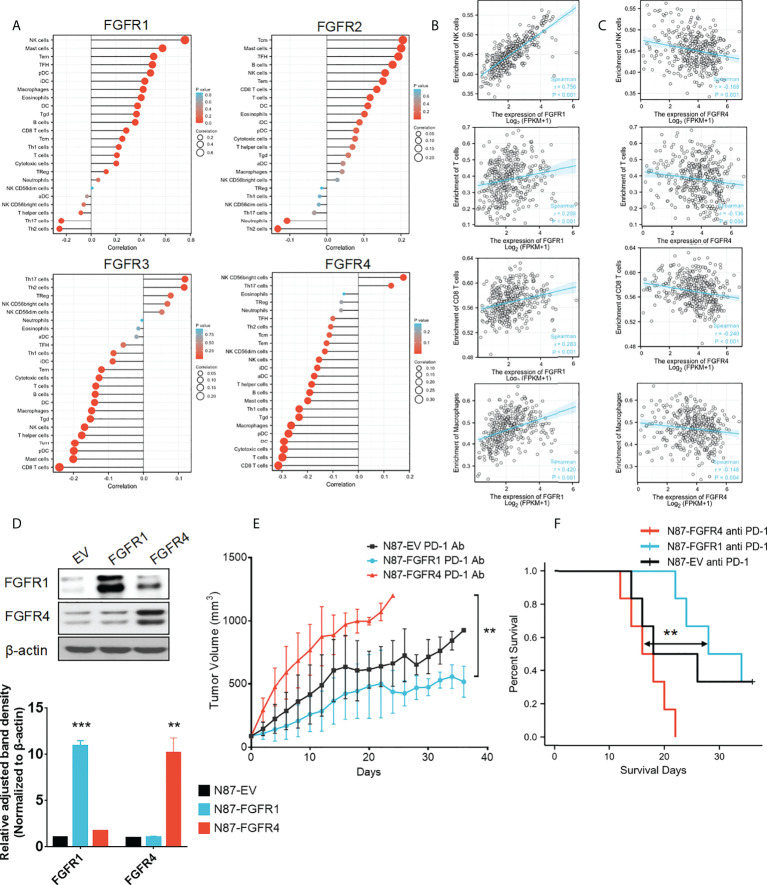
Expression of *FGFRs* related to tumor immune cell infiltration in TCGA-STAD. **(A)** The correlation between FGFRs and the different immune infiltrating cells in TCGA-STAD. **(A, B)** The correlation between FGFR1 **(B)** /FGFR4 **(C)** and NK cell/CD8+ T-cells/B-cells/macrophages in cell infiltration in TCGA-STAD. **(D)** FGFR1 and FGFR4 were stable and overexpressed in NCI-N87 cells. **(E)** The anti-PD-1 mAb was more effective in controlling tumor volume in mice with FGFR1 than FGFR4 overexpressed NCI-N87 xenografts. **(F)** Kaplan–Meier plots of mouse survival. Survival days represents as the time from Day 1 to the day of death or euthanization. **p<0.01, ***p<0.001, log-rank test between groups.

To understand the correlation between *FGFR* family members and immune cell markers, the TCGA-STAD database was investigated using the GSVA package included in the R software. A positive correlation was found between the expressions of *FGFR1* and *FGFR2* in GC patients and the level of immune cell infiltration, which included NK cells, CD8+ T-cells, dendritic cells, and macrophages. However, *FGFR3* and *FGFR4* expressions showed a reverse negative trend (**Figure 7A**). Among all the FGFR family members, the Spearman correlation number between *FGFR1* and NK cells was highest (=0.756). While *FGFR4* had the most obvious negative correlation with the CD8+ T cells (=-0.240). In contrast, the correlation number of *FGFR2* and *FGFR3 wa*s much lower. The correlation between enrichment of distinct immune cells and *FGFRs* are shown in **Figures 7B, C**. TIMER database analysis also revealed a similar pattern (**Figure S6**).

### High expression level of *FGFRs* indicates the different reactions to the anti-PD-1 mAb treatment

To the best of our knowledge, the tumors that could attract more T-cell infiltration are considered as hot tumors, they tend to be more sensitive and effective to immunotherapy. Since we found that *FGFR1* was significantly positively correlated with many immune cells and had a high correlation coefficient, we wondered whether overexpression of *FGFR1* could form a hot tumor, thereby improving the immunotherapy effect. Furthermore, we evaluated whether a negative correlation with *FGFR4* overexpression could diminish the immunotherapeutic consequence. To confirm this, NCI-N87 cells were transfected by pHAGE-FGFR1, pHAGE-FGFR4, and its empty vector. After selecting cells with stable *FGFR* expression, we confirmed their protein levels through a Western blot assay (**Figure 7D**). Then N87-FGFR1, N87-FGFR4, and N87-EV cells were subcutaneously injected into NOG mice. After tumor formation, the mice were randomly allocated to receive either an anti-PD-1 mAb or a placebo. All three kinds of xenograft NOG mouse models showed TV decrease when treated with anti-PD-1 mAb. Among them N87-FGFR1 had most significant differences, while no considerable difference was found in the N87-FGFR4 group (**Figure S7**). In addition, N87-FGFR1 group showed significant regression of average TV after anti-PD-1 mAb treatment compared with the control group, while there was no obvious decrease in the TV of the N87-FGFR4 group compared with the control group when treating with anti-PD-1 mAb. Interestingly, a significant difference was found in mouse bodyweight between the N87-FGFR1 and N87-FGFR4 groups (**Figure 7E**). And the survival analysis revealed that the survival time was also significantly elevated in the N87-FGFR1 group after anti-PD-1 treatment compared with the N87-FGFR4 group (**Figure 7F**). As a result, overexpression of *FGFR1* may improve the immunotherapeutic impact of GC, whereas overexpression of *FGFR4* may impair the immunotherapeutic effect.

### Combination of anti-PD-1 with *FGFR4* inhibitor improves the antitumor immune response in high expression *FGFR4* GC

**Figure 8:**
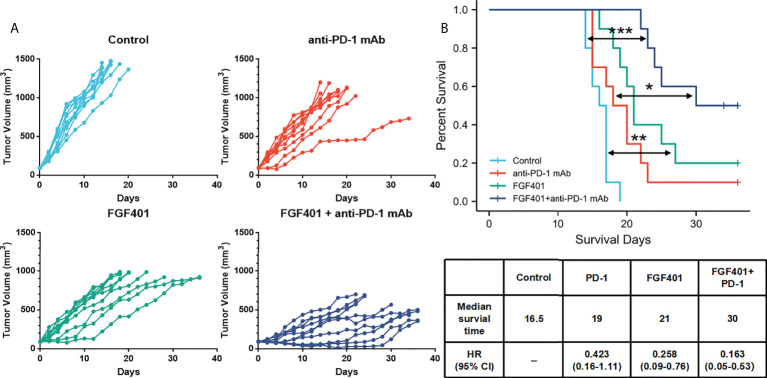
*FGFR4* overexpression in the mouse model re-sensitized to anti-PD-1 mAb treatment in combination with an FGFR4 inhibitor. The N87-FGFR4 subcutaneous xenograft model was divided into different treatment groups either orally treated with FGF401 at 30 mg/kg once day or intraperitoneally injected with anti-PD-1 mAb at 200 mg/mouse twice weekly for 4 weeks, or both. **(A)** TVs of individual mice. Top left: control. Top right: anti-PD-1 mAb group. Bottom left: FGF401 group. Bottom right: FGF401 plus anti-PD-1 mAb treatment group. **(B)** Kaplan–Meier plots of mouse survival. Bottom, a table showed the median survival time of mice in different group and its HR (95% CI). (*p < 0.05; **p<.01;***p < 0.001, log-rank test between groups; TV, tumor volume.

Overexpression of *FGFR4* is not effective for immunotherapy by itself; therefore, a combination of immunotherapy and *FGFR4* inhibitors potentially improve the efficacy of the treatment. We investigated the combination treatment of *FGFR4* inhibitor (FGF401) and anti-PD-1 mAb in an *FGFR4* overexpressed xenograft mouse model. In a mouse subcutaneous xenograft model of N87-FGFR4, the daily anti-PD-1 treatment showed only modest antitumor efficacy compared with the control mice. Following combination with oral dosing of FGF401 at 30 mg/kg, the TV of the mice decreased remarkably (**Figure 8A**). Survival curves of mice in the FGFR4 overexpression model by different treatment (placebo, FGF401, anti-PD-1 mAb, or combination) were presented in **Figure 8B**. The combination of FGF401 and anti-PD-1 treatment significantly prolonged the survival compared to the control group. The similar situation was seen in the FGF401 single-agent group. However, no significant difference was found between the anti-PD-1 mAb group and the control group or FGF401 treatment group. In contrast, mice treated with combination reagents survived longer than mice treated with anti-PD-1 mAb alone (HR, 0.235; 95% confidence interval (CI), 0.07–0.74; *p*= 0.01274). The median survival of the control, anti-PD-1 mAb, FGF401, and combination groups was 16.5 days, 19 days, 21 days, and 30 days, respectively (**Figure 7B**). Collectively, these results demonstrated that a high expression level of *FGFR1* could indicate a good prognosis with the anti-PD-1 treatment, while the opposite was seen with *FGFR4*. Combination therapy with FGF401 and anti-PD-1 could improve the antitumor immune response in high expression FGFR4 GC.

## Discussion

It has been commonly accepted that the FGFR family plays a critical role in biological development (e.g., embryogenesis, angiogenesis, tissue homeostasis) and in regulating physiological processes (e.g., migration, proliferation, and differentiation) (3, 4). Therefore, abnormalities in FGF-FGFR signaling can lead to the development and progression of many cancers, including GC (27–29). Recently, many FGFR related drugs were invented and showed promising result in GC, including pan-FGFR inhibitors, selective FGFR inhibitors, FGFR antibodies and antibody drug conjugates (2). Pemigatinib, the first FGFR1/2/3 selective inhibitor received accelerated FDA approval for patients carrying FGFR2 fusion/rearrangement in cholangiocarcinoma (13). It could also inhibit tumor growth in FGFR2 amplified GC xenograft models. A phase II FiGhTeR trial of pemigatinib in metastatic esophageal-gastric junction/gastric cancer patients with trastuzumab resistant is currently underway (30). Bemarituzumab (FPA144) is a monoclonal FGFR2-IIb isoform-selective antibody. FIGHT trial showed bemarituzumab combined with mFOLFOX remarkably attenuated tumor growth and improved OS of the mestastatic GC patients with FGFR 2b protein overexpression (31). An increasing number of clinical trials of FGFR related drugs are ongoing in GC.

Due to the significant amplification of the FGFR2 gene in gastric cancer, it has become the most popular factor of the FGFR family in GC research (32, 33). However, in the survival analysis of the GEPIA database, no significant differences were found between the FGFR2 mutation-altered group (mainly amplification) and the unaltered one. This may be due to the FGFR2 amplification at the gene level does not lead to the high mRNA levels and protein expression. Furthermore, clinical studies of FGFR family inhibitors in GC have been dominated by inhibition of FGFR2, but their results were largely disappointing (13). Therefore, FGFR2 amplification may not be the best choice in GC for the direction of new drug inventions. Exploring the entire protein family, and in particular other family members and related inhibitors, maybe even more important.

In this study, we explored the expressions of the entire *FGFR* family in GC and their association with clinicopathological characteristics by uncovering TCGA-STAD data with a bioinformatics approach. The expression of *FGFR2* and *FGFR4* was higher in human stomach cancers than in normal tissues, according to the GEPIA and TCGA datasets. While the expression of *FGFR1* and *FGFR3* varied, the differences had no significance in the diverse datasets. The findings in our clinical tissues followed a similar pattern. The data above suggested that FGFRs overexpression might play significant roles in the tumorigenesis and progression of GC, among which *FGFR2* and *FGFR4* varied the most. Our result is consistent with a previous study which showed that the overexpression of *FGFR1*, *FGFR2 and FGFR4* were detected in several GC cases by immunohistochemistry (IHC) staining, whereas *FGFR3* was hardly detectable (34). *FGFR1* (2%), *FGFR2* (< 10%), *FGFR4*(<2%) amplification was frequently reported in the GC sequencing studies (28, 29, 32). *FGFR2* was the most investigated gene in FGFR family. FGFR2 amplification was frequently found in the aggressive diffuse subtype from Lauren’s classification, and associated with lymphatic and venous invasion, lymph node metastasis, distant metastasis, advanced TNM stage, and poor prognosis. However, parts of our results of *FGFR1* differs from results in prior studies. It is possible that our study used a real-time updated database including multiple populations worldwide. But the analysis from previous study using IHC method which is the result from the limited local population. Using the Kaplan–Meier plot, we discovered that an increase in *FGFRs* expression was substantially linked with poor OS and PFS in all the patients with GC who were followed for 180 months. Moreover, The AUC number for *FGFRs* from the ROC curve was high, indicating the diagnostic potential of all *FGFRs* in GC patients. Thus, the high expression of all *FGFR* family members was associated with poor GC prognosis and diagnosis. These results corroborate the findings of many previous studies and a prior meta-analysis, which underscored the clinical and prognostic significance of FGFR1 and FGFR2 overexpression in patients with GC (14, 35). Therefore, *FGFRs* overexpression is a promising diagnostic and prognostic biomarker for GC patients.

Among the entire *FGFR* family, the difference in *FGFR4* expression was more significant. The ROC curve showed the highest AUC for *FGFR4* than any other *FGFR* type. The univariate and multivariate analysis showed that the *FGFR4* expression is the only factor from the *FGFR* family related to the OS and has the potential to be an independent prognostic biomarker. In terms of function, overexpression of *FGFR4* promotes tumor development, cell migration, and invasion. According to the results above, aberrant *FGFR4* plays a significant role in gastric carcinogenesis and could serve as a diagnostic marker and therapeutic target for GC treatment. Although all *FGFRs* have a similar structure, physiological roles, and downstream signaling, *FGFR4* is distinct from the others. *FGFR4* is the only *FGFR* member that is not embryonically lethal when knocked out (36), and its kinase domain is structurally distinct from the others. As a result, *FGFR4* inhibition for cancer therapy may be successful in treating cancer patients with *FGFR4* high expression tumors, causing only minimal side effects. Hepatocellular carcinoma, colon cancer, pancreatic cancer, and breast cancer patients have all been found to have *FGFR4* activation (11, 37–39). However, there are only a few reports of *FGFR4* expression in human GC (40–42). Furthermore, our study showed FGF401(an *FGFR4* inhibitor) could prevent the proliferation of *FGFR4* overexpression in the GC mouse xenograft model. This study supports evidence from preclinical results, that some GC cell lines were sensitive to newly invented FGFR4 inhibitors, such as FGF401, BLU-554 and Futibatinib (43–45). Therefore, FGFR4 inhibitors may be a possible option for future target therapy in GC. In all, our study demonstrated the importance of *FGFR4* high expression in GC survival and provided a new basis for investigating the function of *FGFR4* in GC in the future.

Previous studies on *FGFR* downstream pathways have focused on the RAS/MAPK and PI3K/Akt pathway (40, 46). FGFR regulate *FGFR4* was correlated with GSK-3β in hepatocarcinoma (47). Through GO/KEGG analysis, we found that the enrichment pathways associated with *FGFR4* and *FGFR1* were concentrated not only in the ERK/MAPK and PI3K/Akt pathway, but also ECM receptor pathways. We further validated the crosstalk between *FGFR4* and ERK/MAPK, PI3K-AKT or ECM receptor pathway by *in vitro* experiments. These are consistent with the findings that *FGFR2* mRNA levels were positively associated with Twist-related protein 1 (Twist1), an important transcription factor in the EMT process in the diffuse type of GC. Paulina G et al. also reported that β-catenin and SNAIL were accumulated in the nucleus and associated with resistance of FGFR2 inhibitor (48). For a single target inhibitor, the most probable reason for failure is the arising resistance from the negative feedback loop of its downstream signaling or crosstalk pathway activation. This study revealed that overexpression of FGFR4 activates the ERK/MAPK, PI3K/Akt and ECM receptor pathways, which provides a possible target for the development of combinational treatment strategies. However, our experiments only explored the correlation between the FGFR4 pathway and the PI3K/AKT, ECM receptor pathway, but not the precise regulatory mechanisms and upstream/downstream relationships. Further investigations are needed to explore the detailed regulating mechanisms of *FGFR4* in GC.

Prior studies that have noted the importance of FGF-FGFR axis regulating tumor microenvironment and immune evasion. Wnt1/FGFR1 mice showed significantly enhanced myeloid-derived tumor suppressor cells (MDSC) infiltration and tumor angiogenesis compared to single Wnt1 transgenic mice. The treatment of BGJ398, an FGFR inhibitor, resulted in regression of breast cancer tumors, reduced levels of MDSCs in the surrounding residual stroma, and decreased tumor vascularity (49). Similarly, AZD4547 treatment inhibited proliferation and lung metastasis of mammary tumor cells in mice and reduced MDSCs in the tumor microenvironment and body circulation (50). In our study, we discovered that elevated *FGFR1* expression in GC was positively correlated with tumor immune infiltration and that such overexpressed mouse models were more effectively treated with anti-PD-1 mAb. The opposite correlation was observed in between *FGFR4* expression and immune cell infiltration. And the mouse model with high *FGFR4* expression resulted in insensitivity to immunotherapy. This data is in agreement with several recent studies. Activation of FGFR1 has been shown to induce macrophage recruitment in tumors *via* CX3CL1 induction (51). Jing, W et al. found that inhibition of FGFR3 in bladder cancer to increase PD-L1 protein levels, leading the inhibition of antitumor activity of CD8+ T cells (52). These data show the distinct influence on tumor immune environment of different *FGFRs*, which provide suggestions for prognosis prediction of immunotherapy for GC. Of course, the results need to be confirmed by further clinical evidence and large-scale clinical trials.

Additionally, combining immunotherapy with an *FGFR4* inhibitor could enhance the immune therapeutic effect. These results are in accord with recent investigation indicating that the combination of JNJ-42756493 (erdafitinib), a selective pan-*FGFR* inhibitor, and anti-PD-1 mAb promotes T-cell clonal expansion, and immunologic alterations that enhance antitumor immunity and survival in NSCLC (53). A similar finding was reported by Chenhe Yi et al. in hepatocarcinoma with the combination of lenvatinib and PD-1 mAb (47). A number of clinical trials applying drug combination strategies are currently in process, such as clinical trials were on-going, including erdafitinib with JNJ-63723283 (NCT03473743), nintedanib with ipilimumab and nivolumab (NCT03377023), AZD4547 plus durvalumab (NCT02546661), rogaratinib plus atezolizumab (NCT03473756) and so on (54, 55). But until now, no clinical trial was carried out using a FGFR4 inhibitor plus anti-PD-1 mAb strategy in GC. Further experiments regarding mechanisms and large clinical trials are required to validate the results of our study. It may provide a basis to improve the immunotherapy efficiency in GC.

In conclusion, our study demonstrated that *FGFRs* are overexpressed in GC, and their expression level is associated with the clinicopathological characteristics and prognosis of GC patients, among which *FGFR4* has the most significant diagnostic and prognostic potency. It can be used as a biomarker for diagnosis, treatment, and prognosis of GC. Besides, we also found that the expression level of *FGFRs* was closely related to immune cell infiltration but varied between *FGFR1* and *FGFR4*, which may be a marker for the efficacy of immunotherapy. Combining an anti-PD-1 mAb and an *FGFR4* inhibitor could improve the effect of anti-PD-1 mono-treatment in *FGFR4* overexpression mouse models. This report reveals the critical function of *FGFRs* in GC, as well as the potential connection between *FGFRs* and tumor-immune interactions, which may work as a potential predictor of response to immunotherapy. These findings will help us better understand the function and importance of the *FGFR* family and lay the foundation for further translational and clinical research on the using *FGFRs* and their inhibitors for the diagnosis and treatment of GC.

## Data availability statement

The original contributions presented in the study are included in the article/**Supplementary Material**. Further inquiries can be directed to the corresponding authors.

## Ethics statement

The studies involving human participants were reviewed and approved by the ethics committee of the First Affiliated Hospital of Xi’an Jiaotong University. The patients/participants provided their written informed consent to participate in this study. The animal study was reviewed and approved by the Ethics Committee of the First Affiliated Hospital of Xi’an Jiaotong University.

## Author contributions

All authors were involved in the experiment and editing. CY prepared the figures and wrote the manuscript. SQ and QS critically reviewed and made significant revisions to the manuscript. All authors contributed to the article and approved the submitted version.

## Funding

The present study was supported by the Natural Science Basic Research Plan in Shaanxi Province of China (Program No. 2020JQ*-*494), the Fundamental Research Funds of the First Affiliated Hospital of Xi’an Jiaotong University (Program No. 2017QN-05, 2018QN-03), Institutional Foundation of The First Affiliated Hospital of Xi’an Jiaotong University (Program No. 2022YQPY07) and Clinical Research Award of the First Affiliated Hospital of Xi’an Jiaotong University (Approved ID No. XJTU1AF-CRF-2017-020).

## Conflict of interest

The authors declare that the research was conducted in the absence of any commercial or financial relationships that could be construed as a potential conflict of interest.

## Publisher’s note

All claims expressed in this article are solely those of the authors and do not necessarily represent those of their affiliated organizations, or those of the publisher, the editors and the reviewers. Any product that may be evaluated in this article, or claim that may be made by its manufacturer, is not guaranteed or endorsed by the publisher.
