# Supplementary material for: Comprehensive analysis of the prognostic value and immune infiltration of FGFR family members in gastric cancer
Source: Front Oncol. 2022 Sep 6;12:936952. doi: 10.3389/fonc.2022.936952 (PMC9487308; doi:10.3389/fonc.2022.936952)
Supplement: Supplementary file 1 [file DataSheet_1.pdf]

Supplementary Material

Supplementary Figures and Tables

Supplemental Table 1 Univariate and multivariate COX risk model

Characteristics	Total (N)	Univariate analysis		Multivariate analysis	
		Hazard ratio (95% CI)	*P* value	Hazard ratio (95% CI)	*P* value
T stage	362				
T1	18	Reference			
T2	78	6.725 (0.913-49.524)	0.061	5.528 (0.743-41.136)	0.095
T3	167	9.548 (1.326-68.748)	**0.025**	6.487 (0.887-47.464)	0.066
T4	99	9.634 (1.323-70.151)	**0.025**	5.786 (0.775-43.176)	0.087
N stage	352				
N0	107	Reference			
N1	97	1.629 (1.001-2.649)	**0.049**	1.277 (0.753-2.166)	0.364
N2	74	1.655 (0.979-2.797)	0.060	1.438 (0.833-2.480)	0.192
N3	74	2.709 (1.669-4.396)	**<0.001**	2.262 (1.338-3.826)	**0.002**
M stage	352	2.254 (1.295-3.924)	**0.004**	2.425 (1.326-4.435)	**0.004**
Gender	370	1.267 (0.891-1.804)	0.188		
Age (≤65 *vs* >65）	367	1.620 (1.154-2.276)	**0.005**	1.811 (1.260-2.602)	**0.001**
H pylori infection	162	0.650 (0.279-1.513)	0.317		
FGFR1	370	1.262 (0.908-1.753)	0.166		
FGFR2	370	1.084 (0.780-1.506)	0.631		
FGFR3	370	1.042 (0.751-1.447)	0.804		
FGFR4	370	1.397 (1.006-1.941)	**0.046**	1.431 (1.011-2.026)	**0.043**

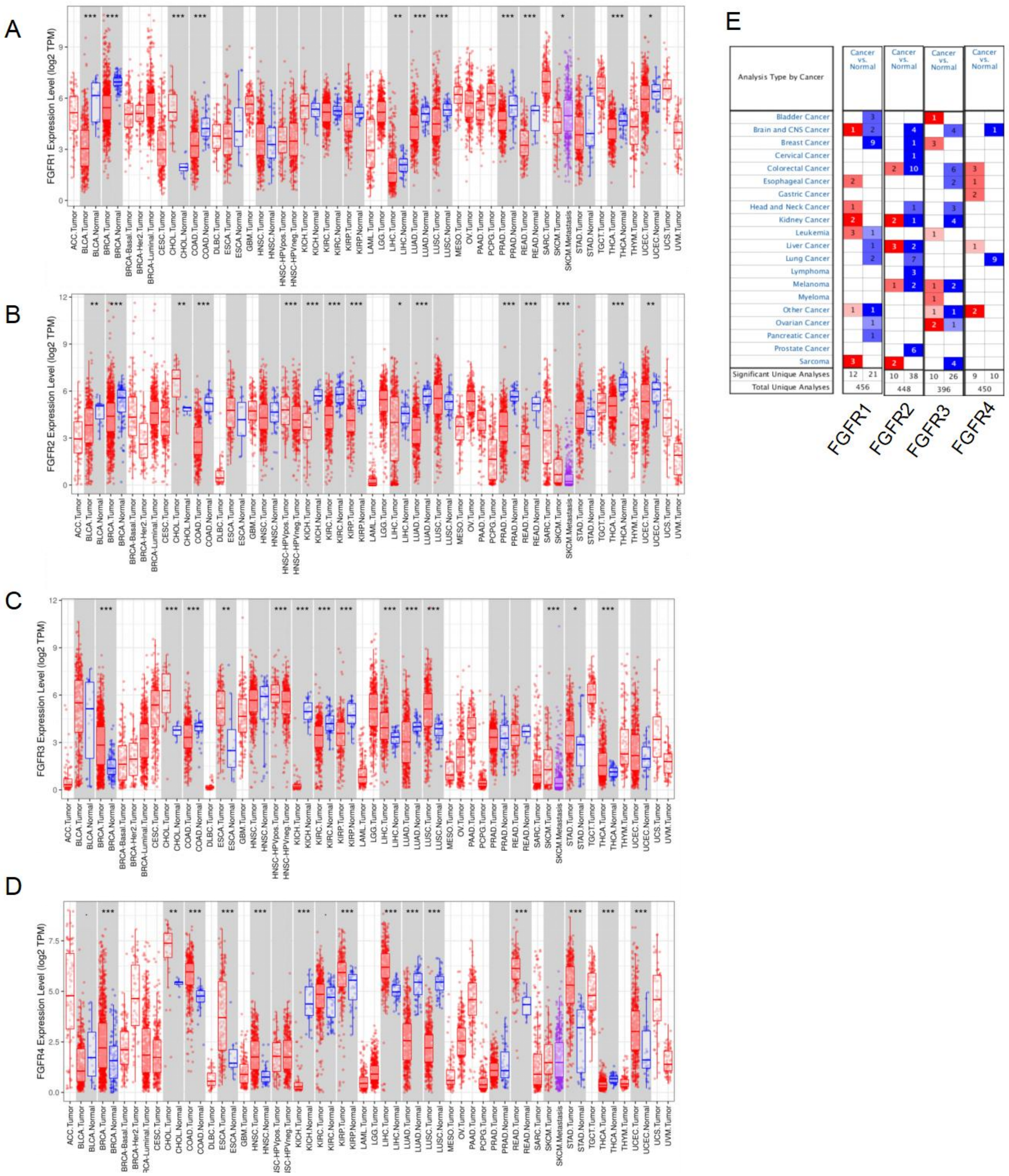

Supplemental Figure 1 *FGFRs* expression levels in different types of human cancers.

(A-D) Expression levels of *FGFR1*(A), *FGFR2*(B), *FGFR3*(C) and *FGFR4*(D) in different tumor types from TCGA database were determined by TIMER. (E) Increased or decreased *FGFRs* in data sets of different cancers compared with normal tissues in the Oncomine database (*p < 0.05, **p <0.01, ***p < 0.001).

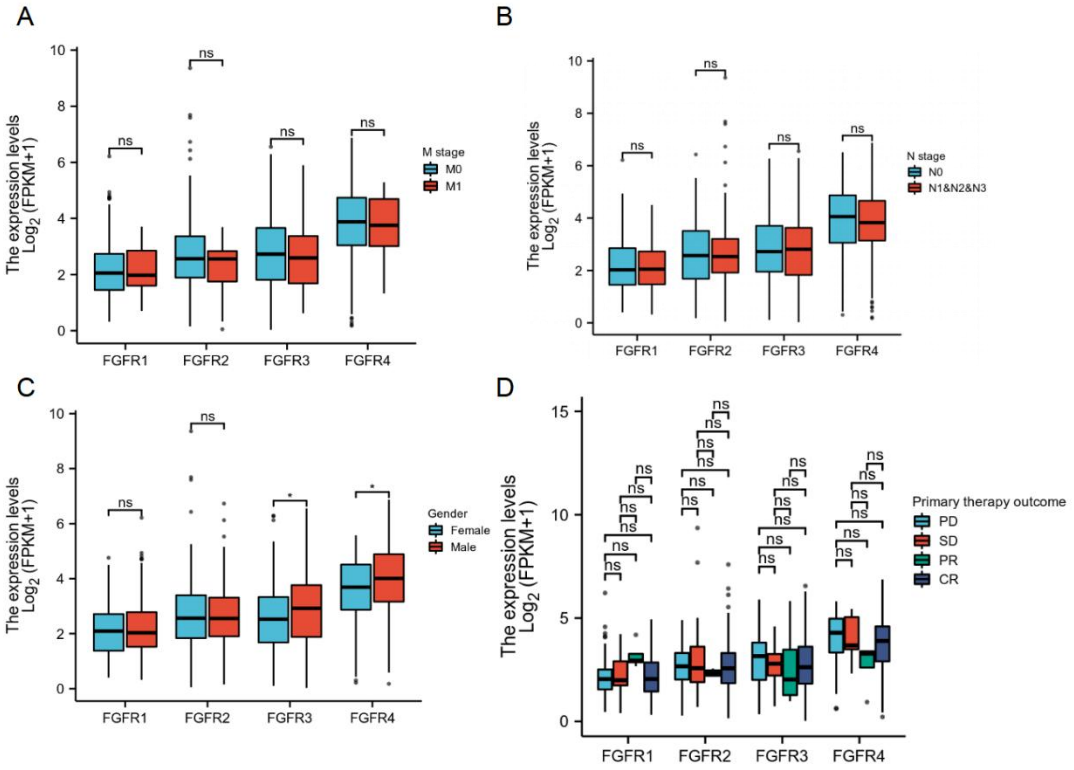

Supplemental Figure 2 Correlation between *FGFR1*(A), *FGFR2*(B), *FGFR3*(C) and *FGFR4*(D) expression and clinicopathological factors in Gastric Cancer Patients (GEPIA).

A: M stage. B: N stage. C: Gender. D: Primary therapy outcome.

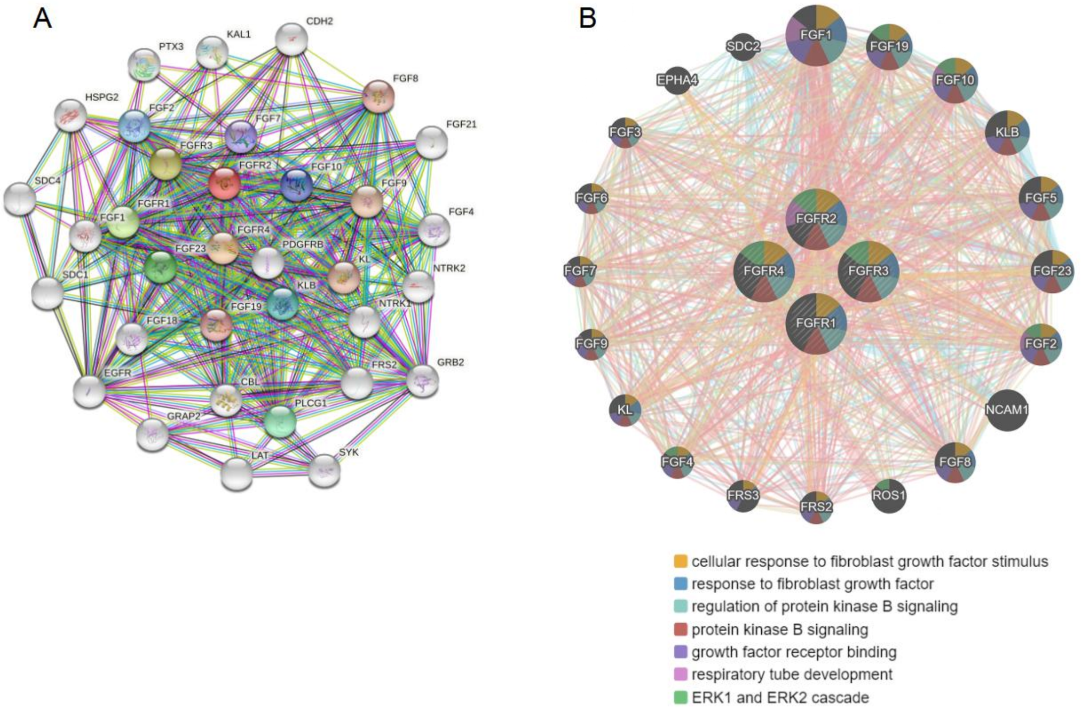

Supplemental Figure 3 Protein–protein interaction network of different expressed *FGFRs* was analyzed by using STRING (A) and GeneMANIA (B).

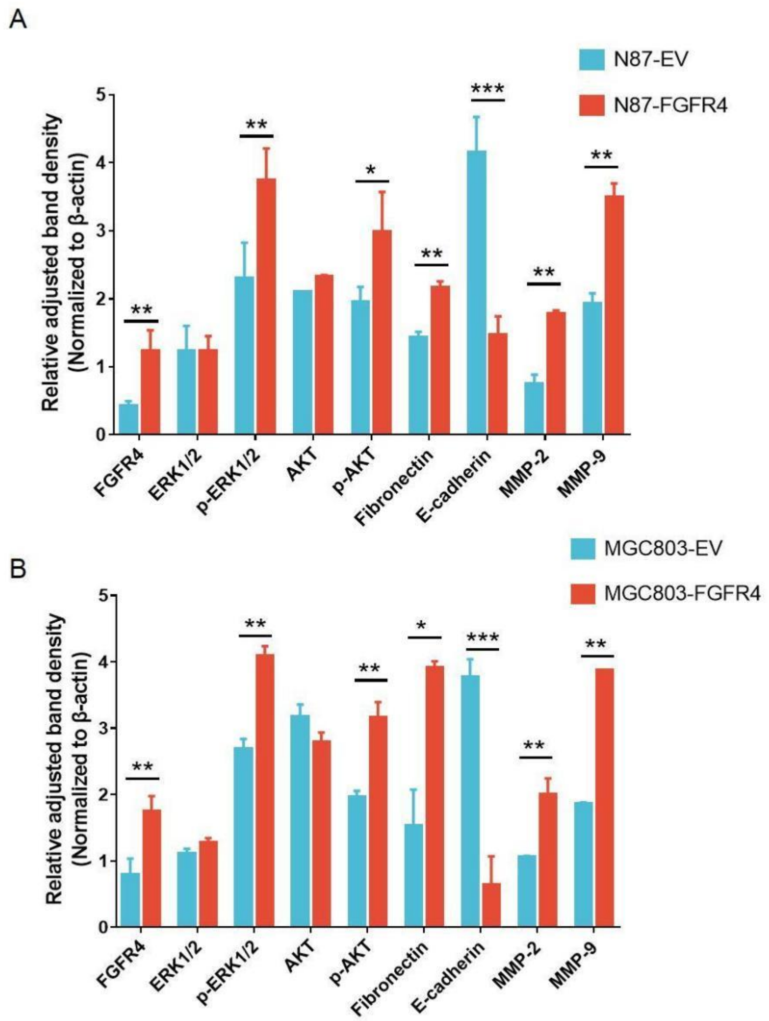

Supplemental Figure 4 The statistical graphs for Western blot experiments in Fig.5F.

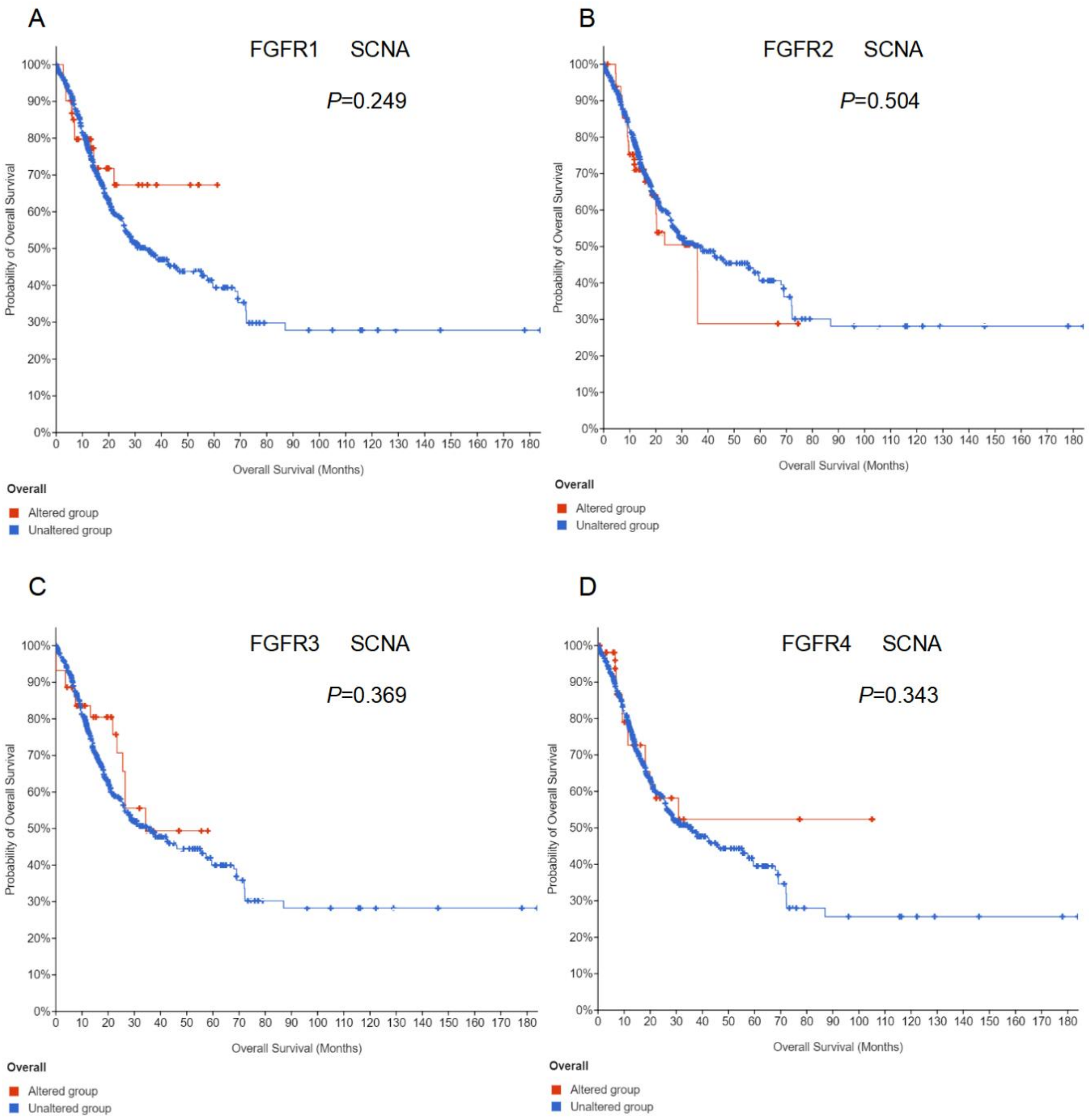

Supplemental Figure 5 The survival curve of *FGFR1*(A), *FGFR2*(B), *FGFR3*(C) and *FGFR4*(D) altered group in STAD through TIMER somatic copy number alternation (SCNA) module.

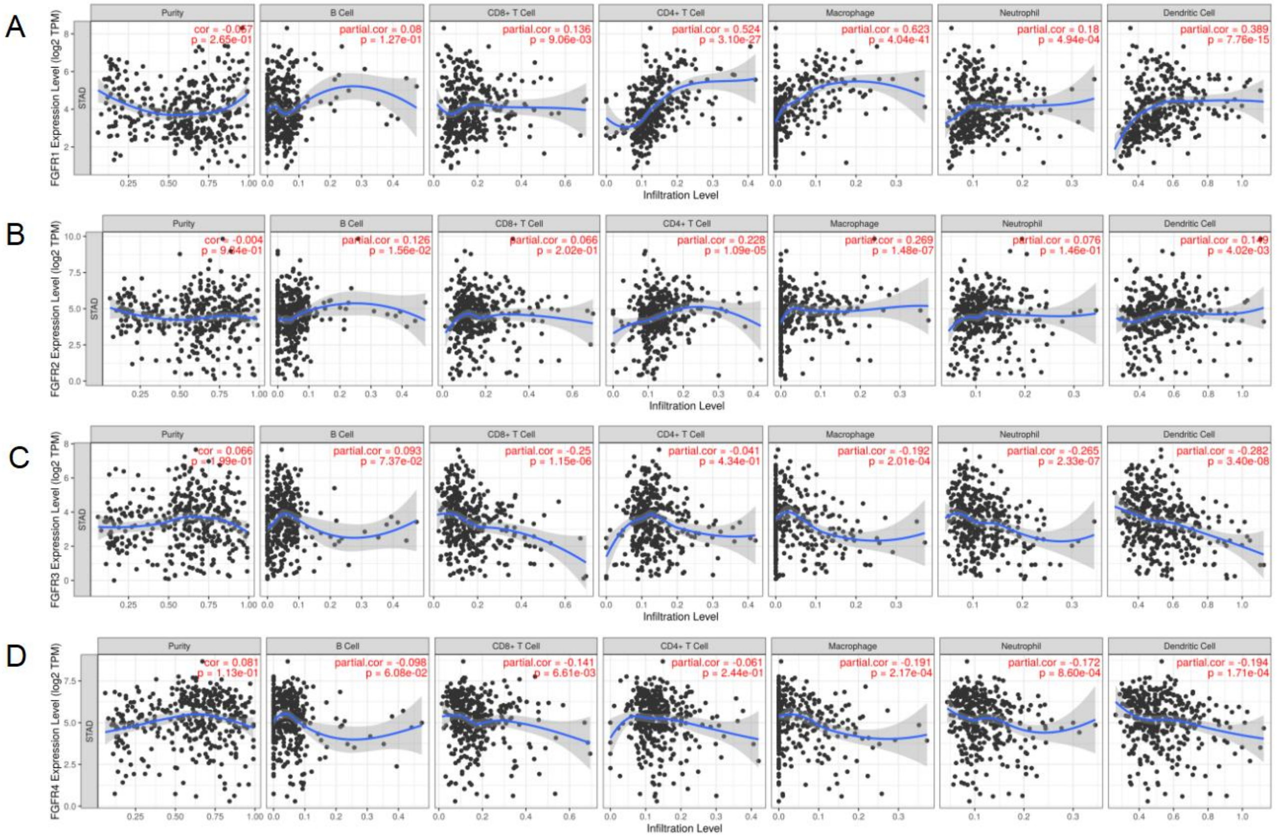

Supplemental Figure 6 Correlation of *FGFR1*(A), *FGFR2*(B), *FGFR3*(C) and *FGFR4*(D) expression with immune infiltration level in STAD.

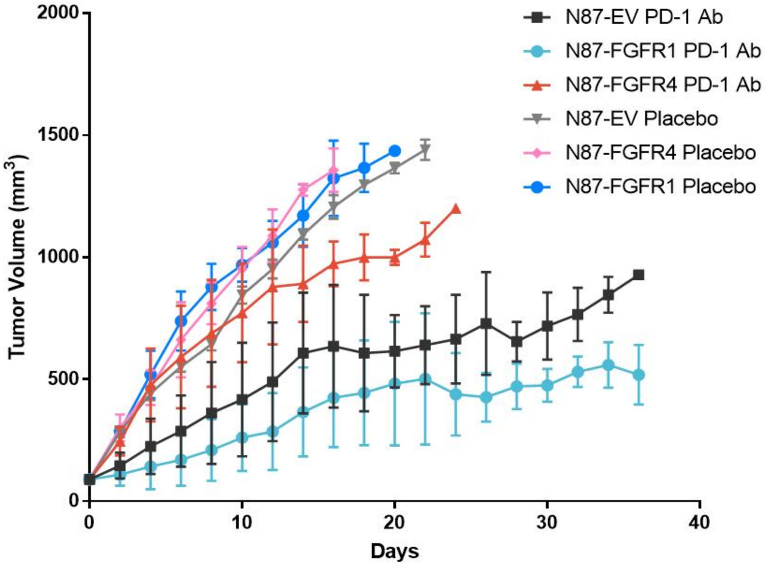

Supplemental Figure 7 The tumor volume in different *FGFRs* overexpressed NCI-N87 xenograft groups treated with anti-PD-1 mAb or placebo.
